# Prevalence and Multilocus Genotyping Analysis of *Cryptosporidium* and *Giardia* Isolates from Dogs in Chiang Mai, Thailand

**DOI:** 10.3390/vetsci4020026

**Published:** 2017-05-10

**Authors:** Sahatchai Tangtrongsup, A. Valeria Scorza, John S. Reif, Lora R. Ballweber, Michael R. Lappin, Mo D. Salman

**Affiliations:** 1 Department of Companion Animal and Wildlife Clinic, Faculty of Veterinary Medicine, Chiang Mai University, Chiang Mai 50100, Thailand; 2 Animal Population Health Institute, Department of Clinical Sciences, College of Veterinary Medicine and Biomedical Sciences, Colorado State University, Fort Collins, CO 80523, USA; mo.salman@colostate.edu; 3 Center for Companion Animal Studies, Department of Clinical Sciences College of Veterinary Medicine and Biomedical Sciences, Colorado State University, Fort Collins, CO 80523, USA; andrea.scorza@colostate.edu (A.V.S.); michael.lappin@colostate.edu (M.R.L.); 4 Department of Environmental and Radiological Health Sciences, College of Veterinary Medicine and Biomedical Sciences, Colorado State University, Fort Collins, CO 80523, USA; john.reif@colostate.edu; 5 Department of Microbiology, Immunology and Pathology, College of Veterinary Medicine and Biomedical Sciences, Colorado State University, Fort Collins, CO 80523, USA; lora.ballweber@colostate.edu

**Keywords:** *Cryptosporidium*, *Giardia*, dogs, Chiang Mai, Thailand

## Abstract

The occurrence and zoonotic potential of *Cryptosporidium* spp. and *Giardia duodenalis* isolated from dogs in Chiang Mai, Thailand were determined. Fecal samples were collected from 109 dogs between July and August 2008. *Cryptosporidium* spp. infection was determined by immunofluorescent assay (IFA), PCR assays that amplify *Cryptosporidium* heat-shock protein 70 kDa (hsp70), and two PCR assays that amplify a small subunit-ribosomal RNA (SSU-rRNA). *Giardia* *duodenalis* infection was identified using zinc sulfate centrifugal flotation, IFA, and four PCR assays that amplify the *Giardia* glutamate dehydrogenase (gdh), beta-giardin (bg), and generic and dog-specific assays of triosephosphate isomerase (tpi) genes. Overall prevalence of *Cryptosporidium* spp. and *G.* *duodenalis* was 31.2% and 45.9%, respectively. Sequence analysis of 22 *Cryptosporidium*-positive samples and 21 *Giardia*-positive samples revealed the presence of *C.* *canis* in 15, and *C. parvum* in 7, *G. duodenalis* Assemblage C in 8, D in 11, and mixed of C and D in 2 dogs. Dogs in Chiang Mai were commonly exposed to *Cryptosporidium* spp. and *G. duodenalis*. *Cryptosporidium parvum* can be isolated from the feces of dogs, and all *G. duodenalis* assemblages were dog-specific. Dogs could be a reservoir for a zoonotic *Cryptosporidium* infection in humans, but further studies will be required to determine the clinical and zoonotic importance.

## 1. Introduction

*Cryptosporidium* spp. and *Giardia duodenalis* are common intestinal protists that can infect humans and animals worldwide [1]. The clinical signs of cryptosporidiosis and giardiasis in dogs vary from sub-clinical to severe diarrhea [2,3].

At least 27 species of *Cryptosporidium* spp. and eight assemblages (A–H) of *G. duodenalis* have been described [4,5]. Although dogs are commonly infected with species-specific *C. canis* and *G. duodenalis* (Assemblages C and D), the occurrence of zoonotic *C. parvum* and *G. duodenalis* (Assemblages A and B) in dogs have raised concern that these animals may serve as a potential reservoir for human transmission [6].

In Thailand, studies regarding cryptosporidiosis and giardiasis in dogs and their zoonotic potential are limited. In one study, *C. canis* was identified in 2 of 95 temple dogs in central Thailand using PCR that amplify an 830-bp fragment of small subunit-ribosomal RNA (SSU-rRNA) gene [7]. The prevalence of *G. duodenalis* infection in temple dogs in the Bangkok area varied from 7.9–56.8% depending on the test used [8,9]. The majority of *G. duodenalis* isolates recovered in these samples were Assemblages A and D. It has been noted that similar genotypes (Assemblage A) were recovered from dogs and humans in the same monastery. In another study in a shelter in Nakornnayok province, the prevalence of *Giardia* infection in shelter dogs using a formalin-ether concentrating technique was 2.8% [10]. To our knowledge, there has been no previous research concerning *Cryptosporidium* spp. and *G. duodenalis* infections and their zoonotic potential in dogs in this area. Since these protist infections are a potential public health concern, determining the prevalence and genotypes of these organisms in dogs living in close proximity to humans and other animals is a priority. Therefore, the aims of this study were to estimate the prevalence of *Cryptosporidium* spp. and *G. duodenalis* infections in dogs in Chiang Mai, Thailand, and to characterize the organism isolates using molecular techniques in order to determine the potential for zoonotic transmission.

## 2. Materials and Methods

### 2.1. Study Location

Chiang Mai is the second largest province of Thailand. It is located in the northern part of the country at geographic coordinates 18°47′ N and 98°59′ E. The city of Chiang Mai maintains its deep roots of traditional community culture in a hybrid landscape of rural and urban city development, and includes agricultural, industrial, and tourism areas. Chiang Mai also represents a tropical environment, which exists in many parts of the world.

### 2.2. Sample Collection

Between July and August 2008, 109 canine fecal samples were obtained from animals visiting the Small Animal Hospital of the Faculty of Veterinary Medicine, Chiang Mai University (*n* = 36), private clinics (*n* = 9), a shelter (*n* = 15), or breeders (*n* = 49) in Chiang Mai province, Thailand. The samples were collected on a volunteer basis regardless of the health status of the animals. Demographic information (age, sex, and housing types) was recorded. Fecal consistency was determined using the Nestle Purina Fecal Scoring System for Dogs and Cats (Nestle-Purina Pet Food Co, St. Louis, MO, USA). Fecal scores of 1–3 were considered as normal, with 4–7 classified as diarrheic.

### 2.3. Determination of *Cryptosporidium* and *Giardia* Infections

*Cryptosporidium* spp. infection was determined using immunofluorescent assay (IFA) and PCR techniques. *Giardia duodenalis* infection was determined using zinc sulfate centrifugal flotation, immunofluorescent assay, and PCR techniques.

#### 2.3.1. Zinc Sulfate Centrifugal Flotation and Microscopy

Fecal consistency was determined upon the receipt of the sample, and all fecal samples were stored in closed plastic containers at 4 °C. Microscopic examination of feces after the performance of a conventional zinc sulfate centrifugal flotation was used to determine intestinal parasitic infection within 5 days of collection, and the remaining fecal samples were stored at −20 °C until being shipped to Colorado State University for IFA and molecular analysis. All fecal samples were shipped to the USA on dry ice and stored at −20 °C until processed.

#### 2.3.2. Fecal Concentration and Immunofluorescent Assay

Prior to IFA and DNA extraction, all fecal samples were concentrated using sucrose gradient centrifugation technique as previously described [11,12]. The IFA slides were processed according to the manufacturer’s instructions (Merifluor^®^ *Cryptosporidium*/*Giardia* IFA kit, Meridian Diagnostic Corporation, Cincinnati, OH, USA). The remaining concentrated fecal material was stored at −20 °C until DNA extraction was performed.

#### 2.3.3. Molecular Detection of *Cryptosporidium* spp. and *Giardia duodenalis* Infection

Three hundred microliters of each fecal concentrate were subjected to DNA extraction following an established protocol [13]. Three PCR assays for *Cryptosporidium* identification were performed. PCR assays amplify a 325-bp fragment of the heat-shock protein (hsp70), a ~290-bp fragment (one-step PCR), and an ~830-bp fragment (nested PCR) of the small subunit-ribosomal RNA (SSU-rRNA) genes were utilized to detect the presence of *Cryptosporidium* spp. [14,15,16]. For *Giardia* molecular identification, four nested PCR assays targeting a 432-bp fragment of glutamate dehydrogenase (gdh), a 510-bp fragment of beta-giardin (bg), and a 511-bp fragment of triose phosphate isomerase genes using generic primers (tpigen) and dog-specific primers (tpiD) were performed as previously described [17,18,19,20]. All PCR assays had several modifications from original publication. PCR mix consisted of 1× HotStarTaq Master Mix (Qiagen, Valencia, CA, USA), 10 pmol of each primer, and 1 μL of template DNA in a final volume of 25 μL for each targeting gene. PCR positive and negative controls were included in every PCR reaction. The *Giardia* positive control was obtained from a dog sample that tested positive for *G. duodenalis* by all four *Giardia* PCR assays, and was subsequently sequenced. The *Cryptosporidium* positive control was obtained from a *C. parvum-*positive cow. The negative control contained the PCR reagents but no DNA. In addition, in nested PCR assays, the negative controls from primary PCRs were included in the secondary PCR assays to evaluate the possibility of contamination.

### 2.4. DNA Sequencing and Genotyping Analysis

The PCR products were evaluated by nucleotide sequencing using a commercially available service (Proteomics and Metabolomics Facility, Colorado State University). The obtained sequences were compared with nucleotide sequences from the nucleotide database from the GenBank by BLAST analysis (http://blast.ncbi.nlm.nih.gov/Blast.cgi).

### 2.5. Data Analysis

A sample was considered positive for *Cryptosporidium* if the sample was positive by either IFA or any of the *Cryptosporidium* PCR assays, and considered positive for *Giardia* if the sample was positive by either zinc sulfate fecal flotation, IFA, or any of the *Giardia* PCRs. Overall prevalence and 95% confidence intervals (CI) were calculated [21]. Associations between *G. duodenalis* or *Cryptosporidium* spp. infections and age (less than one year or one year or more), sex, diarrhea status (yes or no), and housing type (household or breeding kennel/shelter) were assessed using Fisher’s exact test [21]. Odds ratios and 95% CI were estimated using univariate logistic regression analysis to measure the strength of association of each independent variable including age, sex, diarrhea status, housing type, and the presence of co-infection (having both *Cryptosporidium* and *Giardia*). A multivariate logistic regression model against either *Cryptosporidium* spp. or *G. duodenalis* infection in dogs was constructed using a backward stepwise elimination procedure [22]. Variables found to be associated with *Cryptosporidium* spp. or *G. duodenalis* infection in the univariate logistic regression (*p* < 0.25) were included in the multivariable logistic regression analysis. Variables were retained in the model based on the likelihood ratio χ^2^ statistic, at *p* ≤ 0.05. All statistical analyses were performed using the Stata statistical software release 10.1 (Stata Corp., College Station, TX, USA).

## 3. Results

### 3.1. Detection of *Cryptosporidium* spp. and *Giardia duodenalis* Isolates

A single fecal sample was collected from 109 dogs. The characteristics of the samples are shown in Table 1. Fourteen samples (12.8%) were positive for *Cryptosporidium* by IFA; 21 samples (19.3%) were positive by any of the *Cryptosporidium* PCR assays. Thirty-three samples (30.3%) were positive for *Giardia* by fecal centrifugal flotation test; 14 samples (12.8%) were positive by IFA; 21 samples (19.3%) were positive by any of the *Giardia* PCR assays. The overall prevalence of *Cryptosporidium* spp. and *G. duodenalis* infections were 31.2% (95% CI: 22.4–40.0) and 45.9% (95% CI: 36.4–55.4), respectively (Table 2). In addition, in dogs, single infections with *Cryptosporidium* spp. or *G. duodenalis* were 14.7% (16/109) and 29.4% (32/109), respectively. Co-infection of *G. duodenalis* and *Cryptosporidium* spp. was shown in 16.5% (18/109) of the samples.

### 3.2. Genotyping of *Cryptosporidium* spp. and *Giardia duodenalis* Isolates

Eleven sequences from *Cryptosporidium* hsp70, eleven sequences from *Cryptosporidium* one step SSU-rRNA, and eight sequences from *Cryptosporidium* nested SSU-rRNA PCR positive samples were available for genotyping analysis. Using BLAST analyses, 15 dog isolates were typed as *C. canis* and seven were typed as *C. parvum* (Table 3).

Twenty-one sequences from gdh, 18 sequences from bg, 8 from generic tpi, and 15 dog-specific tpi PCR positive samples were available for analysis. Eight dog isolates were typed as *G. duodenalis* Assemblage C, 12 were typed as D, and one C or D depending on target genes (Table 4).

### 3.3. Statistical Analysis

Using χ^2^ or Fisher’s exact tests, age was significantly associated with the prevalence of both *Cryptosporidium* spp. and *G*. *duodenalis* (Table 2). Other variables were not associated with infection.

#### Univariate and Multivariate Logistic Regression Analyses for Risk Associated with *Cryptosporidium* spp. and *Giardia duodenalis* Infection

Univariate logistic regression analyses for categorical variables showed dogs aged less than one year were more likely to be infected with *Cryptosporidium* spp. (OR = 4.10, 95% CI: 1.56–10.76) or *G. duodenalis* (OR = 4.52, 95% CI: 1.61–12.65) than dogs age one year or older (Table 5).

The variables remaining in the model following multivariate logistic regression for *G. duodenalis* infection were age less than one year (OR = 4.11, 95% CI: 1.33–12.70), having diarrhea (OR = 4.59, 95% CI: 1.14–18.49), and residing in breeding kennels or a shelter (OR = 3.723, 95% CI: 1.35–10.26) (Table 6).

## 4. Discussion

The current study represents the first report of the *Cryptosporidium* spp. and *G. duodenalis* prevalence rates and genotypes/species in dogs in Chiang Mai, Thailand. The global prevalence of *Cryptosporidium* spp. and *G. duodenalis* infection in dogs varies depending on the test used, geographic location, and population tested [4,23]. In the present study, overall *Cryptosporidium* spp. and *G. duodenalis* prevalence was 31.2% and 45.9%, respectively. These high prevalences were derived by considering detection in parallel from four tests for *Cryptosporidium* spp. and six tests for *G. duodenalis*.

The prevalence of *Cryptosporidium* spp. is comparable to a previous report of sled dogs from Poland [24], and the prevalence of *Giardia* found in this study is comparable to a previous report of 56.8% in Bangkok [9] and similarly high rates in other countries such as Japan [25], Mexico [26], Brazil [27], Italy [28], and Belgium [29], where most of the studies were from breeding kennels, shelters or abandoned dogs. Nevertheless, the prevalence of these two organisms in this study may have been overestimated due to selection bias. The samples available for this study were not randomly selected, but depended on voluntary participation of the owner visiting the small animal hospital and caregivers of breeders and a shelter. Therefore, the sample may have been biased towards infected and diarrheic animals, resulting in an overestimation of the apparent prevalence.

Zoonotic species or genotypes of *Cryptosporidium* spp. and *G. duodenalis* cannot be distinguished from host-adapted organisms using morphological differentiation. Therefore, molecular characterization using PCR assay and sequence analysis is suggested due to its rapidity and specificity to differentiate the species or genotypes of these organisms. However, not all PCR assays have the same sensitivity for detecting *Cryptosporidium* or *Giardia* nucleotides in fecal samples. In the current study, hsp70 and SSU-rRNA are not in agreement. Of 22 *Cryptosporidium* PCR positive samples, nine were identified from hsp70 only, five from one-step SSU-rRNA only, and two from nested SSU-rRNA only, four from both one-step and nested SSU-rRNA, and two from all three PCRs. It is unclear whether hsp70 or SSU-rRNA have an advantage over each other due to the limitation on the PCR of biological or fecal samples. Of three targeting genes for *Giardia* detection, *Giardia* gdh PCR had the highest amplification rate compared to bg and tpi genes (Table 4). This observation was similar to the study by Scorza and colleagues [12], which showed that the gdh PCR had higher amplification rate than bg or tpi. However, this observation contrasted with the studies by Covacin et al. [30] and Sprong et al. [31], which showed that gdh PCR had the least amplification rate compared to bg, tpi, and SSU-rRNA. In addition, the discrepancies of genotype determination among these three genes have also been reported. Based on this study and our experiences, PCR for the gdh gene may be suggested if the multilocus PCR assay is not affordable.

The majority of genotypes of *G. duodenalis* that infect dogs are host-adapted (Assemblages C or D); however, the pattern can differ geographically [12,4,32]. For example, in the Western United states, zoonotic genotypes of *Giardia* Assemblages A and B were highly prevalent [30], whereas in temple dogs in Bangkok, Thailand, the majority of *Giardia* isolates were identified as Assemblage A [9]. In the current study, all of the *G. duodenalis* isolates were dog-adapted assemblages (C or D). Therefore, the potential of zoonotic *Giardia* transmission from pet dogs in this location is possibly low.

In the current study, from 22 *Cryptosporidium* PCR-positive dogs, 15 specimens were identified as *C. canis* (68%) and 7 specimens (32%) were identified as *C. parvum*. The rate of *C. canis* detection in this study was relevant to previous studies of *Cryptosporidium* isolates from dogs worldwide; 41 *Cryptosporidium* isolates that had been previously reported in dogs, 76% of the isolates were identified as *C. canis*, 22% as *C. parvum*, and 2% as *C. meleagridis* [33,34]. Due to the nature of the cross-sectional study, we are not certain whether the isolated *C. parvum* was a pathogen circulating in the dog population, or transmitted from other animals or humans. The presence of *C. parvum* in the dog samples suggests that dogs could be a potential reservoir for the zoonotic transmission of *Cryptosporidium* spp. While *C. parvum* and *C. hominis* are significant causes of human cryptosporidiosis, the detection of *C. canis* in HIV patients in Thailand [35,36,37] and elsewhere [38,39], as well as the detection of *C. canis* in children [40] have raised concerns regarding the transmission of protozoal diseases from pets to humans even when they harbor the host-adapted pathogens. Further investigation of these parasites among humans and animals living in the same household or in close proximity are needed to confirm this relationship. However, good sanitary practices are highly recommended for all pet owners to avoid zoonotic transmission to humans.

*Cryptosporidium* and *Giardia* genotypes/species isolates from dogs in this study may not reflect the majority of genotypes/species for the dog population as a whole in Chiang Mai, Thailand, since we made our interpretation in the light of the available nucleotide sequences. The information regarding the genotype/species for 35% of *Cryptosporidium-*infected samples and 46% of *Giardia-*infected samples was unknown. Failure of PCR assays to amplify the organisms’ target genes may be from the presence of a PCR inhibitor [41], or degradation of DNA material in the samples which may result from international shipment or long-term storage before PCR processing. Therefore, the failure to amplify *Cryptosporidium* or *Giardia* genes in the fecal samples using PCR did not rule out *Cryptosporidium* or *Giardia* infection. Thus, a PCR should not be used as the primary test for *Cryptosporidium* or *Giardia* clinical diagnosis for practical and cost effective reasons, but it certainly has an important role for confirmation and in research.

Young age, presence of diarrhea, feeding a home-cooked diet, presence of other enteric parasites, being an abandoned or stray dog, and having been kept in a kennel are risk factors that have been reported to be associated with *Cryptosporidium* and *Giardia* in previous studies [42,43,44,45]. Similarly, in this study, *G. duodenalis* infection was shown to be associated with young age, the presence of diarrhea, and coming from a breeder or a shelter. However, to help in prevention and control of these pathogens in dogs in Chiang Mai area, the important risk factors mentioned above including history of the pet’s acquisition, season, and source of drinking water could be applied to this population.

## 5. Conclusions

The current information suggests that the *Cryptosporidium* and *Giardia* infections in young dogs in Chiang Mai are common. Dogs may be a reservoir for zoonotic transmission of *Cryptosporidium*; however, dogs may not be a primary reservoir for zoonotic transmission of *G. duodenalis*. Further investigation using molecular analysis of *Cryptosporidium* and *Giardia* species/genotypes isolated from animals and humans (pets and owners or shelter animals with the caregivers) may clarify the transmission cycle of these organisms between humans and animals in the same environmental setting.

## Figures and Tables

**Table 1:** Characteristics of samples included in the current study (*n* = 109).

Variable	No. of Samples in This Study (%)
*Age*	
*<1 year*	23 (21.1)
*≥1 year*	83 (76.1)
*Unknown*	3 (2.8)
*Sex*	
*Male*	34 (31.2)
*Female*	66 (60.6)
*Unknown*	9 (8.3)
*Diarrhea status*	
*Yes*	17 (15.6)
*No*	89 (81.7)
*Unknown*	3 (2.8)
*Housing type*	
*Breeder and Shelter*	64 (58.7)
*Household*	45 (41.3)

**Table 2:** Prevalence of *Giardia* and *Cryptosporidium* infections by age, sex, diarrhea status, and housing type. Number in parentheses represents the number of samples in each category.

Variable	*Cryptosporidium* spp. % (95% CI *)	*p* Value	*G. duodenalis* % (95% CI *)	*p* Value
Dog (109)	31.2 (22.4–40.0)		45.9 (36.4–55.4)	
Age		0.003		0.003
<1 year (23)	56.5 (34.6–78.4)		73.9 (54.5–93.3)	
≥1 year (83)	24.1 (14.7–33.5)		38.5 (29.9–49.2)	
Sex		0.140		0.666
Male (34)	20.6 (6.3–34.9)		50.0 (32.3–67.7)	
Female (66)	34.8 (23.0–46.0)		45.5 (33.1–57.8)	
Diarrhea status		0.575		0.065
Yes (17)	23.5 (1.0–46.0)		64.7 (39.4–90.0)	
No (89)	32.6 (22.7–42.5)		40.4 (30.1–50.8)	
Housing type		0.392		0.070
Breeder and Shelter (64)	34.4 (22.4–46.3)		53.1 (40.6–65.7)	
Household (45)	26.7 (13.2–40.1)		35.6 (21.0–50.1)	

* 95% CI = 95% confidence interval.

**Table 3:** *Cryptosporidium* genotypes determined by nucleotide sequence analyses of heat shock protein 70 (hsp70), one-step small subunit-rRNA (SSU-rRNA), and nested SSU-rRNA PCR products from dog samples in Chiang Mai, Thailand.

Sample	hsp70	One-Step SSU-rRNA	Nested SSU-rRNA
TH08Dog5	n/a	*C. canis*	n/a
TH08Dog7	n/a	*C. canis*	*C. canis*
TH08Dog22	n/a	*C. parvum*	n/a
TH08Dog28	n/a	*C. canis*	*C. canis*
TH08Dog 42	n/a	*C. canis*	*C. canis*
TH08Dog43	*C. parvum*	n/a	n/a
TH08Dog46	*C. canis*	*C. canis*	*C. canis*
TH08Dog54	*C. parvum*	n/a	n/a
TH08Dog55	*C. canis*	*C. canis*	*C. canis*
TH08Dog58	*C. canis*	n/a	n/a
TH08Dog61	n/a	n/a	*C. canis*
TH08Dog68	n/a	n/a	*C. canis*
TH08Dog69	n/a	*C. canis*	n/a
TH08Dog71	n/a	*C. canis*	n/a
TH08Dog76	*C. parvum*.	n/a	n/a
TH08Dog86	*C. parvum*.	n/a	n/a
TH08Dog87	*C. parvum*.	n/a	n/a
TH08Dog92	*C. canis*	n/a	n/a
TH08Dog96	*C. canis*	n/a	n/a
TH08Dog101	*C. parvum*.	n/a	n/a
TH08Dog102	n/a	*C. canis*	n/a
TH08Dog107	n/a	*C. canis*	*C. canis*

n/a = not available.

**Table 4:** *Giardia* genotypes determined by nucleotide sequence analyses of glutamate dehydrogenase (gdh), β-giardin (bg), and triose phosphate isomerase (tpi) PCR products from dog samples in Chiang Mai, Thailand.

ID	gdh	bg	tpigen ^a^	tpid ^b^
TH08Dog5	D	D	n/a	D
TH08Dog15	D	D	n/a	n/a
TH08Dog17	D	D	C	D
TH08Dog19	C	C	C	C
TH08Dog22	C	C	C	C
TH08Dog23	D	D	n/a	D
TH08Dog24	D^ash^	D	n/a	D
TH08Dog30	C	C	C	C
TH08Dog33	D	D	n/a	D
TH08Dog36	D^ash^	D	n/a	D
TH08Dog40	D	n/a	n/a	n/a
TH08Dog43	D	D	n/a	D
TH08Dog45	D^ash^	D	n/a	D
TH08Dog73	C	C	C	C
TH08Dog93	D	n/a	n/a	n/a
TH08Dog96	D	n/a	n/a	n/a
TH08Dog100	C	C	n/a	n/a
TH08Dog101	C	C	C	C
TH08Dog103	C	C	n/a	n/a
TH08Dog107	D^ash^	C^ash^	C^ash^	D
TH08Dog108	C^ash^	C	C	C^ash^

**^a^** tpi with generic primers; **^b^** tpi with dog specific primers; n/a = not available; ash = allelic sequence heterogeneity.

**Table 5:** Univariate logistic regression analysis of variables associated with *Cryptosporidium* and *Giardia* infections in dogs in Chiang Mai, Thailand.

Variable	Odds Ratio (OR)	95% CI *	*p* Value
*Cryptosporidium* spp.			
Age < 1 year (*n* = 106)	4.10	1.56–10.76	0.004
Sex (male) (*n* = 100)	0.48	0.18–1.28	0.145
Diarrhea (*n* = 106)	0.64	0.19–2.12	0.463
Breeder and Shelter (*n* = 109)	1.44	0.62–3.33	0.393
Presence of *Giardia* infection	1.51	0.67–3.41	0.320
*Giardia duodenalis*			
Age < 1 year (*n* = 106)	4.52	1.61–12.65	0.004
Sex (male) (*n* = 100)	1.20	0.52–2.75	0.666
Diarrhea (*n* = 106)	2.70	0.92–7.96	0.072
Breeder and Shelter (*n* = 109)	2.05	0.94–4.50	0.072
Presence of *Cryptosporidium* infection	1.51	0.67–3.41	0.320

* 95% CI = 95% confidence interval.

**Table 6:** Multivariate logistic regression analysis of variables associated with *Giardia duodenalis* infection in dogs in Chiang Mai, Thailand (*n* = 97).

Variable	Odds Ratios	95% CI *	*p* Value
Age < 1 year	4.11	1.33–12.70	0.004
Diarrhea	4.59	1.14–18.49	0.032
Breeder/Shelter	3.72	1.35–10.26	0.011

* 95% CI = 95% confidence interval.
